# Versatile vapor phase deposition approach to cesium tin bromide materials CsSnBr_3_, CsSn_2_Br_5_ and Cs_2_SnBr_6_†

**DOI:** 10.1039/d0ra04680a

**Published:** 2020-08-03

**Authors:** Sara Bonomi, Maddalena Patrini, Giovanni Bongiovanni, Lorenzo Malavasi

**Affiliations:** Department of Chemistry, University of Pavia, INSTM Viale Taramelli 16 Pavia 27100 Italy lorenzo.malavasi@unipv.it +39 382 987921; Department of Physics, University of Pavia, CNISM Via Bassi 6 Pavia 27100 Italy; Department of Physics, University of Cagliari S.P. Monserrato-Sestu km 0.7 Cagliari 09042 Italy

## Abstract

We report on the successful application of RF-magnetron sputtering to deposit, by using a single type of target, three different materials in the form of thin films within the Cs–Sn–Br compositional range, namely, CsSnBr_3_, CsSn_2_Br_5_ and Cs_2_SnBr_6_. It is shown that, by playing with the deposition parameters and post-deposition treatments, it is possible to stabilize these three perovskites or perovskite related compounds by exploiting the versatility of vapor phase deposition. Full characterization in terms of crystal structure, optical properties and morphology is reported. The power of vapor phase methods in growing all-inorganic materials of interest for photovoltaic and optoelectronic applications is demonstrated here, indicating the advantageous use of sputtering for these complex materials.

## Abstract 2

We report on the application of RF-magnetron sputtering to deposit, by using a single type of target, three different materials in the form of thin films within the Cs–Sn–Br compositional range, namely, CsSnBr_3_, CsSn_2_Br_5_ and Cs_2_SnBr_6_.

## Graphical abstract

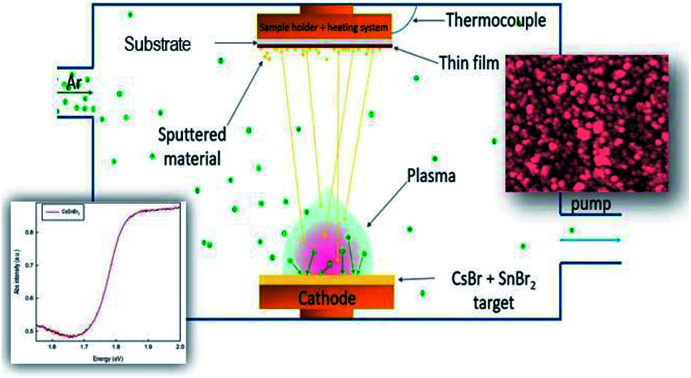

## Introduction

In recent times, there has been a growing interest towards all-inorganic perovskite materials for their application in perovskite solar cells (PSCs) and optoelectronics. Among others, it is possible to mention the use of CsPbBr_3_ and CsPbI_3_, and their solid solutions, in the fabrication of PCSs, as well as their possible use in optical devices due to the superior emission properties when dealing with nanosized materials.^1–5^ Besides the well-established Pb-based all inorganic 3D perovskites, there is an intense and continuous interest in developing lead-free phases together with the search of more stable compositions by reducing the dimensionality of 3D materials, thus exploring 2D, 1D, 0D, and perovskite related phases.^6–10^ 3D lead-free all-inorganic materials are now currently employed in the fabrication of photovoltaic devices, as can be seen by the recent use of CsSnI_3_ or Bi-based double perovkites.^11^ On the other hand, the exploration of lower-dimensional lead-free perovskite and perovskite-related phases is still a challenge. One of the main reason can be found in the difficulty in achieving thin films of all-inorganic materials, where the common wet-chemistry protocols used for hybrid organic–inorganic (HOIP) phases do not reliably assure good results. For example, CsBr has a poor solubility in some apolar solvents such as dimethyl fomamide, DMF, dimethyl sulfoxide, DMSO, *etc.* thus limiting the use of one-step depositions methods.^12^ Even with modified one-step depositions, the methods do not provide uniform films and in other cases annealing at elevated temperatures is required, which is impractical and leads to a reduction of the overall device performance.^13^ Two-step deposition methods provide better results in terms of uniformity but are more time-consuming and require the control of several parameters.^12^

One possible method to overcome such limitation is the use of vapor phase deposition methods, but also in this case the current methods of target evaporation by heating (commonly used for HOIPs) cannot be easily used for inorganic materials showing low volatility. In many cases, after vacuum deposition methods, a thermal annealing at temperature up to 320 °C is required.^14^ In addition to this, when dealing with tin-based systems, one of the most exploited choice to substitute for lead, the stabilization of Sn^2+^ oxidation state during usual solution-based synthetic procedures can be a challenge, thus requiring the use of additives or complex synthetic approaches.^7^

In this paper, we are going to focus on a series of phases, within the Cs–Sn–Br compositional phase diagram, by showing a vapor phase deposition approach based on RF-magnetron sputtering which allows, by tuning the deposition parameters or the post-synthetic treatments, to access at least three single-phase compounds, namely: CsSnBr_3_, CsSn_2_Br_5_ and Cs_2_SnBr_6_, by using the same starting target material. RF-magnetron sputtering has been already shown, by our group, to be a suitable technique for metal halide perovskites because of its benefits in terms of reliability, simplicity, and scalability, among others, In addition, in most of the cases, a fully crystalline and uniform film is obtained without the requirement of any thermal annealing. Quite surprisingly, however, this method has not yet been fully explored in the photovoltaic field.^15^

## Experimental section

### Film deposition

All the thin films have been deposited on amorphous silica substrates (MaTek, roughness *ca.* 1 nm) by means of radio-frequency (RF) magnetron sputtering starting from a CsBr/SnBr_2_ mixture (Aldrich, > 99.9%). The target (diameter 5.08 cm, thickness 1 cm) was made of pressed powders of CsBr/SnBr_2_ mixture. Depositions parameters were: (i) target-to-substrate distance, 8 cm, (ii) RF-power, 50 W and 70 W (iii) argon pressure, 2 × 10^−2^ mbar (iv) argon flux 20 SCCM (v) substrate temperature, 0 °C, 200 °C. The depositions have been carried out in power-control mode. Film thickness has been determined by means of a P-6 stylus profilometer KLA Tencor.

### Post-deposition heating

After the deposition selected films have been heated and cooled in vacuum by using a BÜCHI glass drying oven. Others were instead heated and cooled in air by means of an oven.

### XRD diffraction

The structural properties of the deposited thin films were characterized by X-ray diffraction (XRD) by means of a Bruker D8 Advance instrument (Cu radiation) in a Bragg–Brentano set-up. EDX analysis provided an agreement within 5% between nominal and experimental compositions. Microstructural characterization of the samples was made using a high-resolution scanning electron microscope (SEM, TESCAN Mira 3) operated at 25 kV.

### Optical properties measurement

Absorptance (*A*) spectra were collected by using a UV-vis spectrophotometer Jasco750 with an integration sphere.

### AFM

Atomic Force Microscopy (AFM) images (256 × 256 pixels) were obtained with an AutoProbe CP microscope (ThermoMicroscopes-VEECO), operating in contact mode (C-AFM), by means of sharpened silicon tips onto V-shaped cantilevers (resonance frequency: 15 kHz; force constant: 0.03 N m^−1^). For each analyzed film, scans of 10 μm × 10 μm and 4.0 μm × 4.0 μm have been carried out with a scan rate ranging from 1.0 to 1.5 Hz. A standard second-order flatten processing of the images has been performed to correct the scanner nonlinearity.

## Results and discussion

**Fig. 1:**
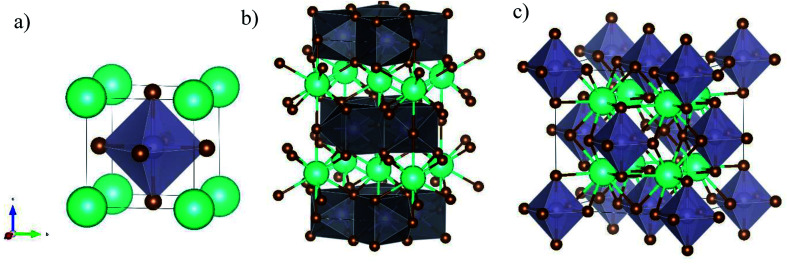
Sketch of the crystal structures of (a) CsSnBr_3_, (b) CsSn_2_Br_5_, and (c) Cs_2_SnBr_6_ showing the peculiar octahedra arrangement in the different structures (see main text for details).

CsSnBr_3_ is a typical 3D perovskite with a cubic unit cell and a band-gap around 1.72 eV, and was object of few studies addressing the growth of films by using spin-coating and reactive thermal deposition to achieve epitaxial materials.^16–18^ CsSn_2_Br_5_ has never been reported in the form of film (except as an impurity phase in ref. 17), and the few data available refer to crystal structure investigation in single crystals.^19^ CsSn_2_Br_5_ has a 2D tetragonal crystal structure belonging to the *I*4/*mcm* space group, and is composed by two adjacent Sn_2_Br_5_ layers separated by a Cs layer along the *c*-axis. Significant work has been carried out on the Pb-based counterpart, namely CsPb_2_Br_5_, which is considered a promising candidate for optoelectronic applications.^20–22^ Finally, Cs_2_SnBr_6_ is a Sn(iv) containing phase of great actual interest for both solar cells and optoelectronic applications, and is a vacancy ordered double perovskite with cubic symmetry (space group *Fm*3̄*m*), and a reported band-gap around 3.2 eV.^23–25^ For this phase no reports on thin film preparation have been reported. A sketch of the crystal structures of the three compounds is reported in Fig. 1.

The samples have been prepared in form of film on fused silica substrates by RF-magnetron sputtering starting from a target made of CsBr and SnBr_2_ (see details in the ESI†). In this paper, we are reporting representative data for the three single-phase compounds obtained, which are the results of several replica of film depositions. As mentioned above, the three compositions have been prepared using a single target by varying the sputtering conditions as shown in Table 1.

**Table 1:** Sputtering conditions used in the experiment. Average thickness of all films was in the range of 800–1000 nm

Phase	Pressure (mbar)	Argon gas flux (sccm)	Power (W)	DC-BIAS (V)	Dep. time	Thermal treatment
CsSnBr_3_	0.02	20	50	90	10 min	200 °C during deposition
CsSn_2_Br_5_	0.02	20	70	150	10 min	—
Cs_2_SnBr_6_	0.02	20	70	120	10 min	200 °C post-deposition

Essentially, the film growth conditions were quite similar for the three phases, with tuning of sputtering power and thermal treatments as key parameters to modulate the phase composition. By heating the substrate during film depositions to 200 °C, CsSnBr_3_ was prepared, while a post-deposition annealing to 200 °C allowed forming Cs_2_SnBr_6_. Without *in situ* or post-deposition heating, CsSn_2_Br_5_ is the stable phase formed under selected conditions.

**Fig. 2:**
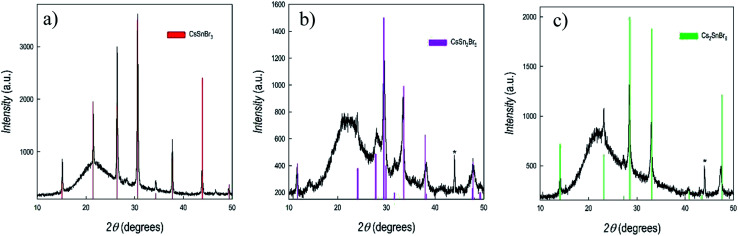
X-ray diffraction patterns of (a) CsSnBr_3_, (b) CsSn_2_Br_5_, and (c) Cs_2_SnBr_6_. Vertical red bars refer to the calculated pattern for each phase. Asterisk indicates reflection from the sample holder.

Fig. 2a–c shows the X-ray diffraction patterns of three films of about 1 μm thickness representing CsSnBr_3_, CsSn_2_Br_5_ and Cs_2_SnBr_6_, together with the reference patterns for each of them (as vertical bars).

As can be seen from Fig. 2, the three films are single-phase with very good crystallinity, also for the sample prepared without any thermal treatment (*i.e*. CsSn_2_Br_5_). No signs of peculiar preferential orientation effects are found in the patterns. Hump around 22° in Fig. 2 is due to the amorphous nature of the substrate (fused silica). Chemical composition of the prepared films was checked by EDX (energy dispersive X-ray analysis) and was found in very good agreement with nominal stoichiometries. The results reported above clearly indicate the versatility of sputtering approach in modulating the deposited phases by simply changing the deposition and/or heat treatment parameters. Being the sputtering a quite complex process, often far from equilibrium, it is not simple, and goes beyond the scope of the present paper, to understand the specific conditions leading to the stability of the different phases, probably related to the different sputtering efficiencies of CsBr and SnBr_2_ and to surface reactions. Notwithstanding, the method is extremely reliable and, by keeping the same sputtering conditions/thermal treatments, the three phases are always obtained. The lattice parameters determined from the refinement of the patterns reported in Fig. 2 are: *a* = 5.8196(7) Å for cubic CsSnBr_3_; *a* = 8.4958(6) Å and *c* = 15.180 (1) Å for tetragonal CsSn_2_Br_5_; and 10.8417(8) Å for cubic Cs_2_SnBr_6_.

**Fig. 3:**
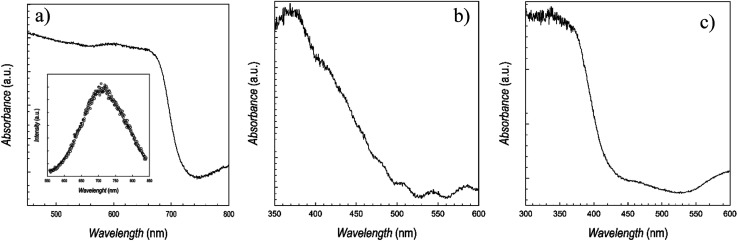
UV-vis absorption spectra of (a) CsSnBr_3_, (b) CsSn_2_Br_5_, and (c) Cs_2_SnBr_6_. Inset of panel (a) reports the photoluminescence spectrum of CsSnBr_3_.

On the representative films shown in Fig. 2, we performed UV-vis absorption spectroscopy measurements, which are shown in Fig. 3, below.

The spectra of CsSnBr_3_ well matches with the data reported for the bulk phase, as well as for the few thin films available, with a very sharp absorbance around 700 nm, and a band-gap of ∼1.73 eV.^16–18,26^ For this material, having and absorption in a range of interest for photovoltaic applications, also the photoluminescence (PL) spectra has been determined, and it is shown in the inset of Fig. 3a, indicating a maximum of emission around 710 nm. This result is also in agreement with previous data.^26^ The quality of the absorption spectra of CsSnBr_3_ film, prepared by sputtering, is significantly higher with respect to the data reported for films prepared by wet-chemistry route, showing edges extending from 400 to 700 nm.^11^ The spectra of CsSn_2_Br_5_ shows broader features, possibly related to the lack of any thermal treatment, with a first edge around 390 nm and a band-gap of about 3.2 eV, in fair agreement with the only available report on this phase, which is however for a very thin film used as a barrier layer.^17^ Finally, Fig. 3c reports the absorption spectra of Cs_2_SnBr_6_ showing again a very sharp edge and an estimated band-gap of about 2.85 eV, in good agreement with previous reports on bulk materials, being this the first time Cs_2_SnBr_6_ is prepared in form of film.^23,24^

**Fig. 4:**
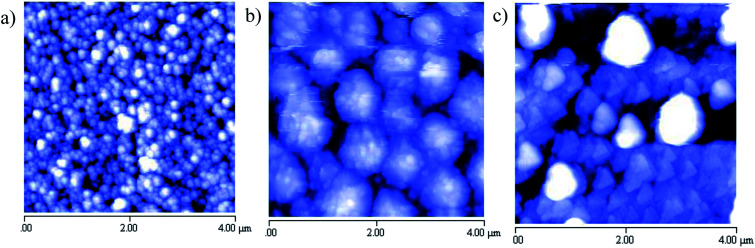
Selected AFM images of (a) CsSnBr_3_, (b) CsSn_2_Br_5_, and (c) Cs_2_SnBr_6_ on 4 μm × 4 μm area.

Finally, the morphology of the three films reported above has been determined by Atomic Force Microscopy (AFM) and some representative images (4 μm × 4 μm area) are shown below (Fig. 4).

There is a markedly different morphology in the three films deposited. CsSnBr_3_ (a) shows well-defined spherical grains of average dimension around 100–150 nm and a surface roughness (*R*_rms_) around 30 nm; CsSn_2_Br_5_ (b) is composed of grains of about 150 nm agglomerated into relatively big spherical objects (500–700 nm) which can be the result of the absence of any thermal treatment; and finally Cs_2_SnBr_6_ films (c) is characterized by polygonal-shaped grains of a size around 200 nm. The peculiar shape of these last grains are in some way reminiscent of the hexagonal form found in nanosized samples of Cs_2_SnBr_6_ particles.^24^ Substrate coverage as well resulted to be quite good for these sputtered films as can be inferred by 10 μm × 10 μm images reported in the ESI.† Unfortunately, there are not direct AFM images collected on analogous film to perform any relevant comparison. It is interesting to note, however, that any specific composition leads to a peculiar morphology (for analogous film thicknesses) (Fig. 4).

## Conclusions

This paper reports the successful deposition of three distinct single-phase materials in form of thin films of the Cs–Sn–Br system by RF-magnetron sputtering, namely CsSnBr_3_, CsSn_2_Br_5_ and Cs_2_SnBr_6_. The deposition approach used in this work allowed using the same starting target material and tuning the preparation of the desired phase by changing the sputtering parameters or applying mild post-deposition heat treatments. Structural, optical and morphology measurements confirm the quality of the prepared films. The accessibility of complex all-inorganic phases, which may be difficult to deposit in form of films by means of traditional wet-chemistry routes, is demonstrated through a simple, reliable and scalable vapor-phase method such as sputtering.

## Conflicts of interest

There are no conflicts to declare.

## Supplementary Material

RA-010-D0RA04680A-s001
