# Supplementary material for: Versatile vapor phase deposition approach to cesium tin bromide materials CsSnBr3, CsSn2Br5 and Cs2SnBr6
Source: RSC Adv. 2020 Aug 3;10(48):28478–82. doi: 10.1039/d0ra04680a (PMC9055831; doi:10.1039/d0ra04680a)
Supplement: RA-010-D0RA04680A-s001 [file RA-010-D0RA04680A-s001.pdf]

Electronic Supplementary Material (ESI) for RSC Advances.
This journal is © The Royal Society of Chemistry 2020

ELECTRONIC SUPPLEMENTARY INFORMATION

Versatile Vapor Phase Deposition Approach to Cesium Tin Bromide Materials $CsSnBr_3$, $CsSn_2Br_5$ and Cs_2SnBr_6

Sara Bonomi,[a] Maddalena Patrini,[b] Giovanni Bongiovanni,[c] Lorenzo Malavasi[a,]*

[a]Department of Chemistry, University of Pavia and INSTM, Viale Taramelli 16 Pavia, 27100, Italy;

[b]Department of Physics, University of Pavia and CNISM, Via Bassi 6 Pavia, 27100, Italy;

[c]Department of Physics, University of Cagliari, S.P. Monserrato-Sestu km 0.7 Cagliari, 09042, Italy

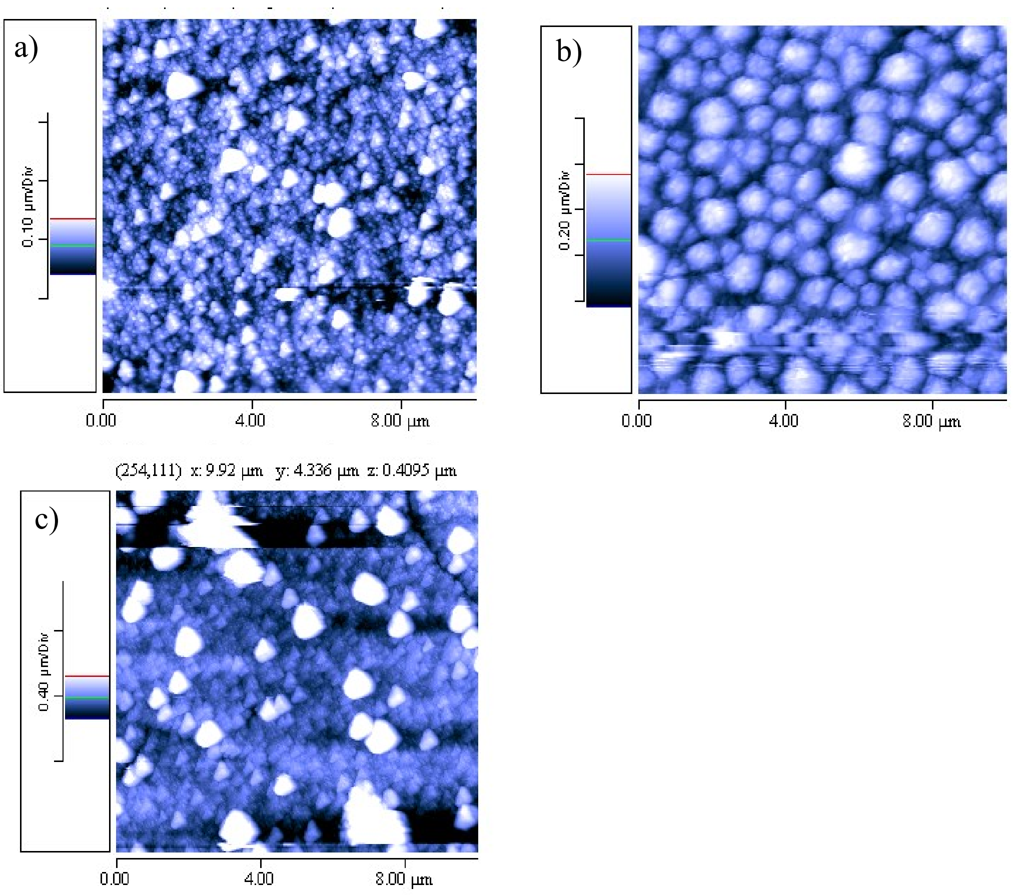

Figure 1: Selected AFM images of a) $CsSnBr_3$, b) $CsSn_2Br_5$, and c) Cs_2SnBr_6 on 10 µm × 10 µm area.
